# Esophagitis in a High *H. pylori* Prevalence Area: Severe Disease Is Rare but Concomitant Peptic Ulcer Is Frequent

**DOI:** 10.1371/journal.pone.0025051

**Published:** 2011-10-11

**Authors:** Julio Ponce, Xavier Calvet, Marta Gallach, Marta Ponce, and the Esophagitis Study Group of the Asociación Española de Gastroenterología (AEG)

**Affiliations:** 1 Servicio de Aparato Digestivo, Hospital Universitari La Fe, Valencia, Spain; 2 CIBEREHD Instituto de Salud Carlos III, Barcelona, Spain; 3 Institut Universitari Parc Taulí, Departament de Medicina, Universitat Autònoma de Barcelona, Barcelona, Spain; 4 Unitat de Malalties Digestives, Hospital de Sabadell, Sabadell (Barcelona), Spain; Charité-University Medicine Berlin, Germany

## Abstract

**Background:**

Few data are available on the prevalence of erosive and severe esophagitis in Western countries.

**Objective:**

To retrospectively determine the prevalence and the factors predicting erosive esophagitis and severe esophagitis in a large series of endoscopies in Spain.

**Design:**

Retrospective observational study. A multivariate analysis was performed to determine variables predicting severe esophagitis.

**Setting:**

Databases of 29 Spanish endoscopy units.

**Patients:**

Patients submitted to a diagnostic endoscopy during the year 2005.

**Interventions:**

Retrospective review of the databases.

**Main Outcome Measurements:**

Esophagitis severity (graded according to the Los Angeles classification) and associated endoscopic findings.

**Results:**

Esophagitis was observed in 8.7% of the 93,699 endoscopies reviewed. Severe esophagitis (LA grade C or D) accounted for 22.5% of cases of the disease and was found in 1.9% of all endoscopies. Incidences of esophagitis and those of severe esophagitis were 86.2 and 18.7 cases per 100,000 inhabitants per year respectively. Male sex (OR 1.89) and advanced age (OR 4.2 for patients in the fourth age quartile) were the only variables associated with severe esophagitis. Associated peptic ulcer was present in 8.8% of cases.

**Limitations:**

Retrospective study, no data on individual proton pump inhibitors use.

**Conclusions:**

Severe esophagitis is an infrequent finding in Spain. It occurs predominantly in males and in older individuals. Peptic ulcer disease is frequently associated with erosive esophagitis.

## Introduction

Prevalence of symptoms of gastro-esophageal reflux disease (GERD) is uniformly high in Western countries. Studies on GERD prevalence show that between 10% and 20% of the Western general population report GERD symptoms (reflux, regurgitation or both) at least once a week [1]–[5]. Studies performed in Spain showed similar prevalences of frequent GERD symptoms ranging from 9.8% [6] to 12% [7]. Although little information is available, two longitudinal series estimated that the yearly incidence of newly diagnosed GERD ranges from 4.5% to 5.4% [8], [9]. Much less is known about the frequency of endoscopic lesions and, specifically, of severe esophagitis. Studies suggest that when endoscopy is performed to study reflux symptoms erosive esophagitis is found in approximately 50% of patients [10]–[14]. Similarly, in a recent US study approximately 60% of volunteers with frequent heartburn participating in a clinical trial had esophagitis. In that study, prevalence of severe disease was unexpectedly high: 29.6% of patients had grade A or B and 25.8% grade C or D esophagitis [15] on the Los Angeles classification [16]. By contrast, studies in the general population suggest a yearly incidence of erosive esophagitis of 15 to 25 per 1000 persons/year, approximately ten times lower than that of occasional GERD symptoms [17], [18].

In addition, management of acid-related diseases has been modified by the widespread availability of proton pump inhibitors (PPI) both as prescription and over-the-counter drugs. Empirical treatment of heartburn before endoscopy is currently the rule and it is well known that the prevalence of endoscopic findings is sharply reduced by previous antisecretory treatment [19]. It would be interesting to determine whether the diagnosis of severe esophagitis remains frequent under the current conditions of widespread PPI use.

Finally, to our knowledge, there are no published studies estimating the prevalence of erosive esophagitis in the Mediterranean area. For all these reasons, the present study was designed to retrospectively assess the current prevalence of peptic esophagitis, and especially of forms of severe disease, from the records of a large sample of Gastrointestinal Endoscopy Units in Spain.

## Methods

### Ethics Statement

The study was reviewed and approved by the Ethics Committees of both the Hospital Universitari La Fe and the Hospital de Sabadell. A written informed consent was obtained from all participants involved in the study. Data were coded in order to avoid patient identification.

### Study Design

**Figure 1:**
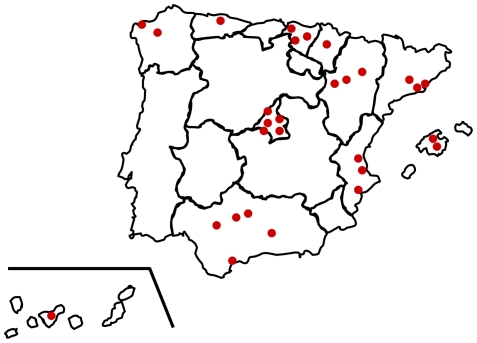
Geographical distribution of participating centres.

Thirty-five Gastrointestinal Endoscopy Units were invited to participate in the study, and twenty-nine accepted (figure 1). The main reason for declining participation in the study was the lack of an electronic database allowing easy data retrieval. General information was provided on the endoscopy unit, including the number of upper gastrointestinal endoscopies performed in 2005 and the rate of endoscopies ordered by general practitioners. In addition, the hospital level and the population served were obtained from the Annual Administrative Reports of the participating hospitals for 2005. Data from the upper endoscopies performed from January to December 2005 were retrieved from the databases of the different units. The number and demographic characteristics (age and gender) of patients with endoscopic diagnosis of peptic esophagitis, the severity of the disease – graded according to the Los Angeles (LA) classification system [16] – and the associated endoscopic findings were specifically recorded. Severe esophagitis was defined as LA grade C or D lesions. Data were recorded in two MS Excel databases – one for the hospital and general data, and the other for information on the individual patient.

Demographic data for the reference populations of the different hospitals for 2005 were retrieved from Spanish government databases [20]. Finally, in an attempt to determine indirectly whether the use of antisecretory drugs could influence the rates of the endoscopic diagnosis of esophagitis, data on the regional sales of these drugs were compared with the rates of esophageal disease in the area. Data on drug sales were kindly provided by IMS Health S.A. (2005 antisecretory drugs sales in Spain, IMS Health S.A:. Madrid-Spain).

### Statistical analysis

The prevalence of peptic esophagitis and severe esophagitis were calculated for the total number of endoscopies. In addition, age was divided into ten-year intervals and the prevalence of esophagitis and severe esophagitis was determined for each age interval. Yearly incidence of esophagitis was also calculated. Mann-Whitney U test was used for comparing quantitative variables to the occurrence of esophagitis and severe esophagitis. Additionally, a multiple logistic regression model was performed to identify factors associated with the occurrence of esophagitis. SPSS 11 was used for the analyses.

### Sample size calculation

The study was designed to evaluate the prevalence of severe esophagitis with a confidence interval of ±1%. Extrapolating from the literature, we assumed that 25% of all esophagitis would be severe and that esophagitis would be found in 40% of all endoscopies. The estimated minimal number of endoscopies to analyze was 5972.

## Results

### Number of endoscopies and referral population

The 29 participating hospitals were widely distributed throughout Spain (figure 1). They provided medical assistance for a total population of 10,431,252 individuals, corresponding to 23.6% of the entire Spanish population. The mean referral population was 324,536 subjects per hospital, ranging from 107,000 to 787,000 subjects. Twenty-two institutions were high-level teaching hospitals and 7 were county hospitals. 93,699 upper endoscopies were performed during 2005, a rate of 0.89 endoscopies per 100 habitants per year.

### Number of patients with esophagitis and severe esophagitis

Esophagitis was reported in 8,189 endoscopies (8.7% of all those performed during 2005). Esophagitis patients' age ranged from 15 to 105 years (mean 53.5±18 years). Sex distribution was: 5649 male and 2540 female. According to the LA classification [16], esophagitis severity was graded as A in 3754 patients (45.8%), B in 2595 (31.7%), C in 960 (11.7%) and D in 880 (10.8%) (table 1). Severe peptic esophagitis (LA C–D) was, therefore, detected in 22.5% of patients, representing 1.9% of all endoscopies.

**Table 1:** Severity of esophagitis according to sex.

			LA classification				Total
			A	B	C	D	
SEX	Male	n	2326	1917	738	668	5649
		%	41.2	33.9	13.1	11.8	100
	female	n	1428	678	222	212	2540
		%	56.2	26.7	8.7	8.4	100
Total		n	3754	2595	960	880	8189
		%	45.8	31.7	11.7	10.8	100

**Figure 2:**
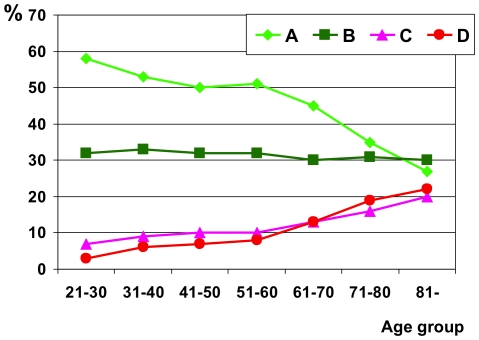
Severity of esophagitis according to age.

Peptic esophagitis was more prevalent in males (69%). Severe esophagitis was also mainly diagnosed in males and its prevalence increased at advanced age; nearly 40% of the esophagitis in patients over 70 years were LA C and D (figure 2).

Annual incidence of endoscopic esophagitis was 86.2 cases per 100,000 habitants per year. Incidence of severe esophagitis (LA grade C or D) was 18.7 cases per 100,000 habitants per year.

A logistic regression was performed to ascertain the risk increase for severe esophagitis associated with increasing age and male sex. Both variables were found to be independently related to the development of severe esophagitis, with the risk increasing geometrically with age with an OR of 4.2 in the patients in the fourth quartile. OR was 1.89 for males (table 2). Regression was validated using the ROC curve method. Area under the curve was 0.663 with a p value lower than 0.001.

**Table 2:** Results of the multivariate analysis.

	Sig.	Exp(B)	95% C.I.for EXP(B)	
Age (quartiles)			Lower	Upper
14–39	,000	1		
40–52	,000	1.408	1.176	1.686
53–68	,000	2.018	1.701	2.394
69–105	,000	4.223	3.584	4.977
Male sex	,000	1.889	1.660	2.148

### Associated endoscopic findings

Esophagitis associated to esophageal or gastric malignancy was unusual: 27 (0.3%) and 74 (0.9%) endoscopies respectively. By contrast, gastro-duodenal peptic disease was very frequently associated with reflux esophagitis. Thus, duodenal ulcer was present in 5.6% of endoscopies, gastric ulcer in 3% and combined gastric and duodenal ulcers in 0.2%.Erosive duodenitis was present in 9.6% and gastric erosions in 7.7% of patients with esophagitis. Overall, 8.8% of patients had a peptic ulcer and 22.4% of patients had some kind of peptic lesion, many having more than one type of lesion. Both peptic ulcer (10% vs. 5.8%) and peptic lesions (24.6% vs. 18.8%) were significantly more frequent in males. In addition, prevalence of both duodenal and gastric ulcer increased with age, from 3.4% and 0.8% in patients in their twenties to 7.4% and 5.3% in patients over 80 years respectively. By contrast, erosive gastritis and duodenitis remained more stable, increasing only from 5.3% to 7.4% and from 5.1% to 7.9% respectively.

### Risk factors related to severe esophagitis

Prevalence of severe esophagitis was not related to hospital level (15.6 cases per 100,000 habitants for county hospitals vs. 14.8 cases for 100,000 habitants for reference hospitals), or to the rate of endoscopies indicated by general practitioners, the number of inhabitants in the reference area, the mean age of the population or the regional expenditure on PPI.

## Discussion

Our study suggests that, in Spain, peptic esophagitis is unusual (8.7%) in patients who undergo upper endoscopy for a variety of upper gastrointestinal symptoms. Severe esophagitis is even less frequent, appearing in fewer than 2% of endoscopies. Moreover, the figure of 18 cases per 100,000 patients-years yearly incidence of severe esophagitis is one of the first estimates of the incidence of this disease in a Mediterranean country.

In addition, our study confirms the results of published studies suggesting that advanced age [21]–[25] and male sex [11], [17], [23], [25], [26] are important risk factors for severe erosive esophagitis. The combination of male sex and advanced age produces an almost tenfold increase in the risk of severe esophagitis. There is no complete explanation for these findings. In the case of age, it is reasonable to hypothesize that the age-related decrease in esophageal sensitivity could allow more severe lesions to develop before medical advice is sought. This hypothesis is in concordance with other studies in which elderly subjects had a significantly lower prevalence [21] and severity [22] of typical gastroesphageal reflux symptoms. However, not all data, support this hypothesis. In a study reported only in abstract form, Fennerty et al [15] observed that the prevalence of severe esophagitis was higher in young patients (age <65 years). The data from this last study should, however, be considered with care since the subjects recruited were volunteers participating in a clinical trial, with the result that the risk of selection bias was high.

Another interesting finding of our study is that the prevalence of associated peptic ulcer disease in patients with an unequivocal diagnosis of GERD was almost 10% and that peptic erosions were found in more than 20% of the endoscopies. As countries in the Mediterranean area like Spain have a high prevalence of *Helicobacter pylori* infection [27] and both peptic ulcer and esophagitis are frequent diseases, the association of the two conditions is also expected to be frequent. The finding of a very high prevalence of gastroduodenal ulcer in patients with typical reflux symptoms or esophagitis has also been observed in other areas where *Helicobacter pylori* infection is highly prevalent, for example in Asia [25], [28], [29]. Taken together these data suggest that, in areas with a high prevalence of *Helicobacter pylori* infection, empirical PPI treatment for suspected GERD may be inadequate for a significant proportion of patients since *Helicobacter pylori*-related peptic ulcer disease is often an associated finding. In consequence, *Helicobacter pylori* should probably be investigated and treated to rule out peptic disease before assigning the patient to chronic PPI therapy for clinically – or even endoscopically – diagnosed GERD.

A major strength of the study is that the total referral population - over 10 million individuals - represents nearly a quarter of the Spanish population. In addition, data were obtained from 29 hospitals from all over the country (figure 1). These factors contribute to reduce the risk of local biases and support the reliability of the data.

This study has, however, several methodological limitations inherent in its retrospective design. First, the criteria for diagnosing esophagitis or establishing their severity had a variable interobserver rate that depends on the scoring system used and the level of experience of the endoscopist [30]–[32]. However, it has been reported that agreement between observers is better in more severe degrees of disease [33]. It is likely, therefore, that the accuracy of the data on the prevalence of severe esophagitis was acceptable.

A second limitation is that, although a tentative incidence of severe esophagitis was estimated, the study is not population-based and the data should be treated with care. Some local factors, however, suggest that the estimate is reliable. In Spain, the health care system has universal coverage and the proportion of private practice is low. In fact, public coverage of endoscopy is over 90% in most of the areas and cross-referrals between different areas for endoscopy are unusual. In addition, the data were consistent: for example, despite the difference in complexity, the rate of severe esophagitis did not differ between referral and County hospitals.

A third possible limitation (one which applies to all studies estimating esophagitis prevalence) is that selection criteria for endoscopy will vary between different age groups as well as between different areas. The prevalence of different gastrointestinal lesions varies depending on the symptoms investigated. The prevalence of esophagitis has been reported to range from 6% to 25% in dyspeptic patients [26], [34] and from 55% to 65% in patients with gastroesophageal reflux symptoms [15], [23]. Therefore, the prevalence will be strongly influenced by the policy for indicating endoscopy. This could explain the high variability of reflux esophagitis reported in different studies, ranging, for example, from 7% to 15% in the general Japanese population [11], [18]. In any case, as severe esophagitis is associated with more severe and frequent symptoms [23], the likelihood of missing severe disease is probably far lower for severe than for mild esophagitis.

A final limitation of the study is that it was not possible to obtain data on antisecretory drug use previous to the endoscopy. Antisecretory drug use is rising continuously. For example, in a Scandinavian study, Lassen et al [35] reported an increase in the already high use of antisecretory drugs before endoscopy (33% in 1993 and 41% in 2002). The influence of PPI use on the prevalence of severe esophagitis is difficult to evaluate. As stated above, endoscopic studies suggest that acid-reducing treatment before endoscopy cures most peptic lesions and limits its usefulness for detecting esophagitis or peptic ulcer [19]. It could therefore be inferred that the prevalence of esophagitis and severe disease will decrease as long as PPI use increases. However, the evidence is scarce: only the Kalixandra study evaluates previous treatment with antisecretory drugs [18]. Even in this case, data are difficult to extrapolate to the current situation, as the study dates from 1998, when the use of antisecretory drugs was low. In addition, the authors did not attempt to correlate PPI use and the presence or absence of esophagitis.

In conclusion, our study suggests that, at present, severe esophagitis is not a frequent finding. Severe endoscopic disease predominates in males and the elderly. In addition, peptic ulcer disease and erosive gastro-duodenitis are common associated findings. This stresses the importance of ruling out peptic ulcer, preferably by non-invasive testing followed by *Helicobacter pylori* eradication before establishing long-term PPI treatment in countries with a high prevalence of this infection. Additional studies will be necessary to determine the influence of antisecretory drug treatment in the prevalence of endoscopic esophagitis.
